# Impact of Vanadium Complexes Treatment on the Oxidative Stress Factors in Wistar Rats Plasma

**DOI:** 10.1155/2011/206316

**Published:** 2011-10-11

**Authors:** R. Francik, M. Krośniak, M. Barlik, A. Kudła, R. Gryboś, T. Librowski

**Affiliations:** ^1^Department of Bioorganic Chemistry, Medical College, Faculty of Pharmacy, Jagiellonian University, 9 Medyczna Street, 30-688 Krakow, Poland; ^2^Department of Food Chemistry and Nutrition, Medical College, Faculty of Pharmacy, Jagiellonian University, 9 Medyczna Street, 30-688 Krakow, Poland; ^3^Medical College, Faculty of Pharmacy, Jagiellonian University, 9 Medyczna Street, 30-688 Krakow, Poland; ^4^Faculty of Chemistry, Jagiellonian University, 9 Ingardena Street, Krakow, Poland; ^5^Department of Pharmacodynamics, Medical College, Faculty of Pharmacy, Jagiellonian University, 9 Medyczna Street, 30-688 Krakow, Poland

## Abstract

The aim of this study was to investigate the clinical efficacy of vanadium complexes on triglycerides (TG), total cholesterol (Chol), uric acid (UA), urea (U), and antioxidant parameters: nonenzymatic (FRAP—*ferric reducing ability of plasma*, and reduced glutathione—GSH) and enzymatic (glutathione peroxidase—GPx, catalase—CAT, and GPx/CAT ratio) activity in the plasma of healthy male Wistar rats. Three vanadium complexes: [VO(bpy)_2_]SO_4_·2H_2_O, [VO(4,4′Me_2_bpy)_2_]SO_4_·2H_2_O, and Na[VO(O_2_)_2_(bpy)]·8H_2_O are administered by gavage during 5 weeks in two different diets such as control (C) and high fatty (F) diets. Changes of biochemical and antioxidants parameters are measured in plasma. All three vanadium complexes statistically decrease the body mass growth in comparison to the control and fatty diet. In plasma GSH was statistically increased in all vanadium complexes-treated rats from control and fatty group in comparison to only control group. Calculated GPX/CAT ratio was the highest in the control group in comparison to others.

## 1. Introduction

In physiological condition Reactive Oxygen Species (ROS) play an important role as mediators and metabolism regulators. ROS can stimulate the glucose transport to the cells, they can be secondary messengers in the growth and death of cells. Increase of ROS and parallel exhaustion of antioxidative reserve is known as antioxidative stress. Various chronic diseases, such as inflammatory diseases, cancer, cardiovascular or metabolic diseases, are associated with an increased oxidative stress, a process characterized by an excessive formation of simple, highly reactive molecules, or ROS, such as superoxide anions, hydrogen peroxides, or peroxynitrites [1, 2]. This is why the development of tests aimed at determining the relativeness between the production of ROS and the antioxidant properties in the organism has become a clinical issue, especially since treatment with antioxidants is suspected to have a preventive effect on pathogenic processes. 

Diabetes, especially type 2, is one of more common diseases in highly developed countries [3, 4]. The dynamics of development of this disease suggests that in the future more people can have problem with glucose tolerance. As the first step of treatment of this disease, diet and life style can be sufficient but not for a long time. After this period, medical treatment is necessary. At present, there are a lot of medicaments in the diabetes treatment but new molecules, which will be more positive than the present ones, are searched for. Usually in patients with diabetes type 2, we can observe a lower level of natural antioxidants in blood such as ascorbic acid, tocopherol, reduced glutathione, and uric acid [5]. 

From the 1980s vanadium and its compounds have been tested as new medicaments in diabetes. For this moment organic complexes have more interesting properties than the inorganic compounds. Vanadium has also the ability to change the oxidative step in a living organism and can have a positive or negative influence on total the oxidative defense. This mechanism is very variable and dependent on the oxidative step, used dose, type of ligands, presence of vitamin C, tocopherol, and others. As oxidative stress may play a role in the development of many diseases, different antioxidants are useful in their treatment. Among various antioxidants vanadium complexes seem to be promising [6]. Vanadium is one of the trace elements really existing for a living organism. Vanadium-organoligand complexes have been used to examine the structure and activity of many proteins, taking opportunity of spectroscopic techniques [7]. There are naturally appearance ligands that bind vanadium, such as the iron binding siderophores. Siderophores are involved in iron homeostasis, and their binding of vanadium occurs to be a secondary function [8]. Vanadate does inhibit the transport of iron-siderophore complexes [9], showing that there is an interaction of vanadium with iron transport systems. Many natural metabolites, including glutathione, cysteine, ascorbic acid, nucleotides, and carbohydrates, form complexes with vanadium that have been characterized by the different authors in [10–12]. The interactions of vanadium and antioxidant like reduced glutathione or superoxide dismutase, a particularly well-defined system, could be intimately involved in the interactions of cellular vanadium and redox properties [13]. Because oxidative stress may play role in the development of many diseases, different antioxidants are useful in the treatment of them. Among the various antioxidants, vanadium complexes seem to be promising [6]. 

In this work, the animal model with control and high fatty diet (30% w/w of saturated fats acids) has been used in the diabetes treatment, high fatty diet with small portion of carbohydrates is frequently proposed. This work shows changes in the chosen biochemical and antioxidant parameters (TG, Chol, UA, U, FRAP, GSH, GSHt, GPx, CAT, and GPX/CAT ratio) in Wistar rats after vanadium complexes administration by gavage in both the control and fatty diet. These parameters play an important role in oxidation defense, and it is important to study the influence of this metal on homeostasis in tested animals.

## 2. Materials

### 2.1. Vanadium Complexes

Vanadium complexes used for this experiment have been synthesized by Dr. R. Grybos from the Faculty of Chemistry of Jagiellonian University in Krakow. Bipyridine and methyl bipiperidine was used as ligand but differences between these complexes were associated with the oxidative step of vanadium (vanadium IV and vanadium V). Tested complexes. In the present work, three complexes were used: [VO(bpy)_2_]SO_4_·2H_2_O marked as B, [VO(4,4′Me_2_bpy)_2_]SO_4_·2H_2_O-marked as Bm, and Na[VO(O_2_)_2_(bpy)]·8H_2_O marked as V.

### 2.2. Animals

The experiment was conducted on the 3-month-old male Wistar rats, weighing 250 ± 15 g and caged in the temperature of 23°C, humidity 50–60%, and light-dark cycle (12/12 h). Each group consisted of 6 animals. During the time of the experiment, the C group was fed with the standard diet (Table 1), while the C+V, C+B, and C+Bm groups were fed with the standard chow with the addition of vanadium complex V, vanadium complex B, and vanadium complex Bm, respectively. The animals from the F group received the highfatty diet (Table 1) The animals from the F+V, F+B, and F+Bm groups received the fatty chow with the addition of vanadium complex V, vanadium complex B, and vanadium complex Bm. In all vanadium treated groups, tested complexes were administered by gavage once a day during 5 weeks in the dose of 20 mg/kg body mass. The experiments were performed in accordance with legal requirements, under a license granted by the Local Commission of Ethics in Krakow. After 5 weeks of the experiment the animals were anesthetized, and blood was collected from the abdominal aorta. The blood was centrifuged during 15 min 3000 r/min and frozen until the analysis.

## 3. Methods

### 3.1. Biochemical Analysis

Biochemical analysis was made with the standard biochemical analyzer Alize with standard kits (Chol, TG, UA, and U) from Biomérieux, and it was controlled with Control Serum 1, ODC0003 and Control Serum 2, ODC0004 (OLYMPUS). All the reagents were of analytical grade and were obtained from Sigma Aldrich Chemical Company (Steinheim, Germany).

### 3.2. Measurement of FRAP Activity

The FRAP method has been used in antioxidant properties measurements. In acidic environment, Fe^3+^ present in FRAP is reduced to Fe^2+^, possessing intensive blue colour, with maximum absorbance at 593 nm. This reaction undergoes with any substance, which exhibits reduction properties. The FRAP is the modification of Benzie and Strein's [14] method. In case of the FRAP method, the Fe^2+^ content in the tested samples of plasma was calculated based on the standard curve. The FRAP concentration values (mM) one read for the tested substances of 15 min.

### 3.3. Measurement of Glutathione Peroxidase (GPx) Activity

Glutathione peroxidase GPx (EC 1.11.1.9) activity in the rat plasma was assayed with Paglia and Valentine's method (Paglia and Valentine 1967), using H_2_O_2_ and NADPH as substrates. The conversion of NADPH to NADP^+^ was followed by recording the changes in absorption intensity at 340 nm, and one unit was expressed as 1 nM of NADPH consumed per minute/mg protein

### 3.4. Measurement of Catalase (CAT) Activity

Catalase CAT (EC 1.11.1.6) activity was measured with Aebi's method [15] and estimated in plasma. The measurements were performed spectrophotometrically at 240 nm at 25°C. One unit of CAT activity was defined as the amount of enzyme decomposing 1 *μ*mol of H_2_O_2_ per minute. CAT concentrations were expressed in U/mg of protein. Balance between antioxidant enzymes was expressed as a ratio between glutathione peroxidase and catalase (GPx/CAT ratio). GPx/CAT ratio was calculated after the measurement of catalase activity according to Aebi's method.

### 3.5. Statistical Analysis

The results in this study were expressed as the means and standard deviations SDs. Differences in the results between the studied subjects were analyzed with the ANOVA test. Statistical analyses were performed with STATISTICA PL software, version 8.0 (Statsoft Pl).

## 4. Results and Discussion

**Figure 1:**
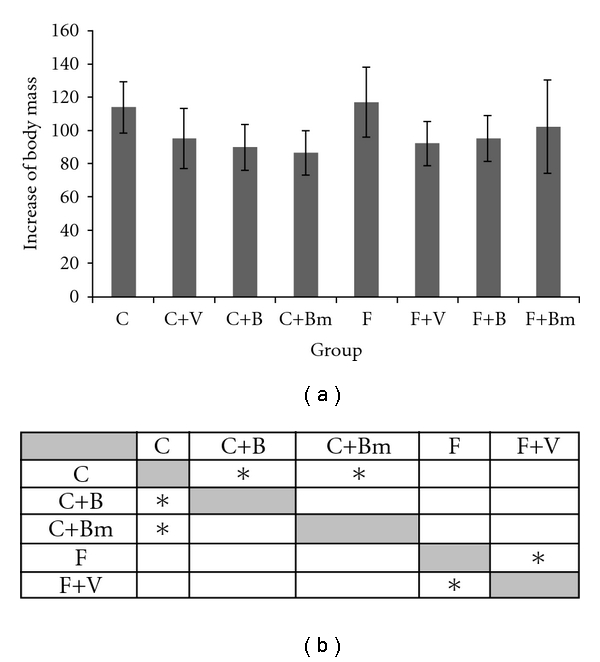
Values of animal weight changes of animals fed with a standard diet and high fatty diet (*P* ≤ 0.2005) with the addition of vanadium complexes tested. Data presented as mean ± SD for *n* = 6. (b) Statistical differences between groups *P* < 0.05.

The effect of vanadium complexes on changes in increase of body weight is shown in Figure 1. It was observed that the addition of vanadium compounds to the standard diet caused reduced weight gain. In case of complex B, weight gain was 89.83 ± 13.8 (*P* = 0.0455; Figure 1(a)) and in group Bm 86.3 ± 13.3. in comparison to control group. These changes were statistically significant and accounted for about 25% reduction in weight gain (*P*  = 0.0027; Figure 1(a)). Complex V, added to the high fatty diet, also caused a statistically significant (*P*  = 0.0455), decreased weight gain (92 ± 13.1 g) in comparison to high fatty diet. The vanadium complexes, depending on the applied diet (diet C or diet F), caused the reduced increase of body mass.

**Figure 2:**
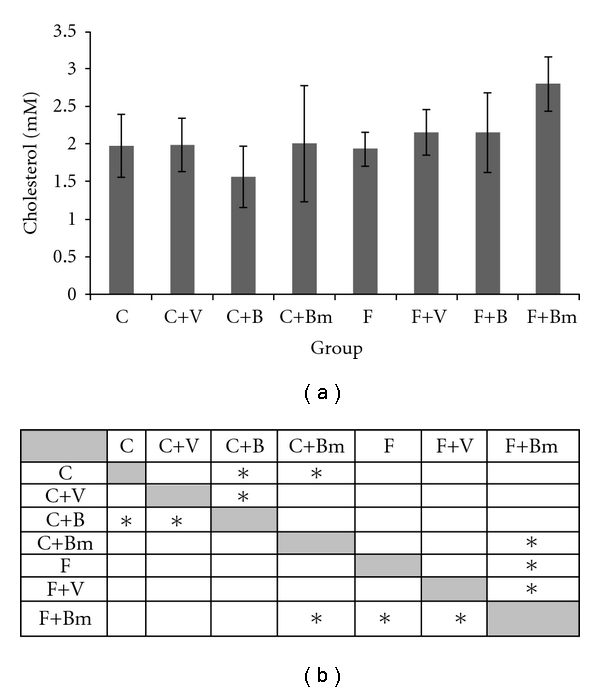
Effects of vanadium complex V, B, and Bm on plasma of total cholesterol of Wistar rats. Data are presented as mean ± SD for *n* = 6. (b) Statistical differences between groups *P* < 0.05.

Figure 2 shows changes in total cholesterol levels. Vanadium complex B, given in the diet C, caused a reduction of cholesterol levels by about 21%. Complex Bm added to the diet F caused an increase of cholesterol levels by 45% (*P*  = 0.0027; Figure 2(a)). Other groups showed no statistically significant effect on the change in cholesterol levels. 

**Figure 3:**
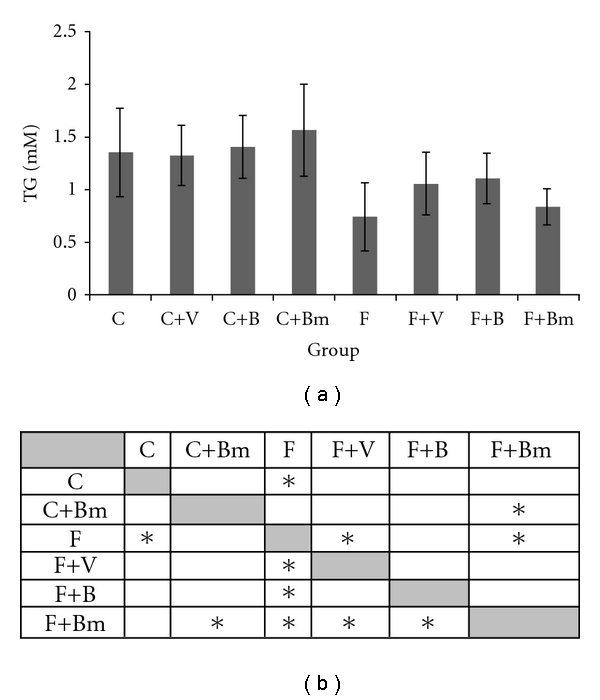
Effects of vanadium complex V, B, and Bm on plasma triglycerides (TG) of Wistar rats. Data are presented as mean ± SD. for *n* = 6. (b) Statistical differences between groups *P* < 0.05.

For TG levels (Figure 3), there was no significant change in the group of animals fed with the control diet. However, the vanadium complexes V and B added to the F diet caused increased concentration of TG. In the F group, TG concentration was 0.75 ± 0.3 mM, while in the F+V group 1.06 ± 0.29 mM and in the F+B group 1.11 ± 0.24 mM. 

**Figure 4:**
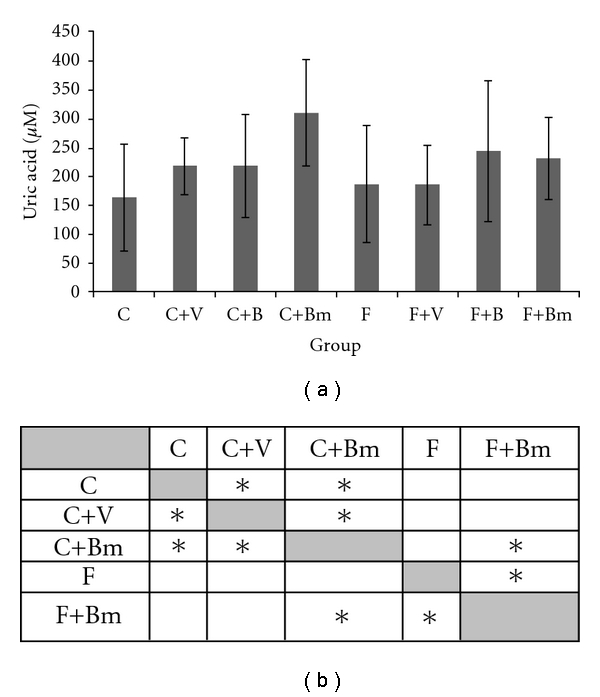
Effects of vanadium complex V, B, and Bm on plasma uric acid of Wistar rats. Data are presented as mean ± SD for *n* = 6. (b) Statistical differences between groups *P* < 0.05.

The supply of vanadium complexes (V, B, and Bm) caused an increase in uric acid levels (Figure 4) in animals with control diet. Vanadium complexes V and Bm caused a statistically significant increase in uric acid levels (*P* = 0.0455 and *P* = 0,0027; Figure 4(a)). In groups of animals fed with the F diet, the addition of vanadium compounds B and Bm caused a statistically significant increase in uric acid levels (*P*  = 0.0455; Figure 4(a)). 

**Figure 5:**
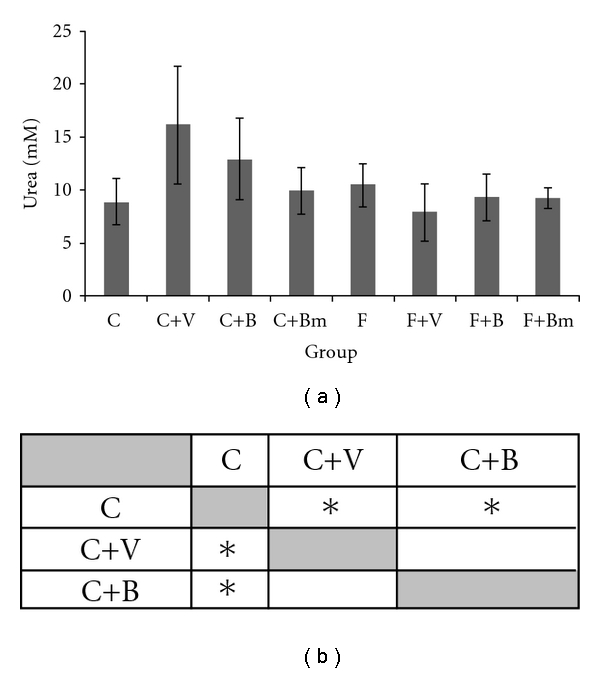
Effects of vanadium complex V, B, and Bm on plasma urea of Wistar rats. Data are presented as mean ± SD for *n* = 6. (b) Statistical differences between groups *P* < 0.05.

Figure 5 presents the impact of vanadium complexes on the concentration of urea. In animals fed with the C diet, the addition of complex V or B caused a significant increase in the concentration of this parameter (*P*  = 0.045; Figure 5(a)). 

**Figure 6:**
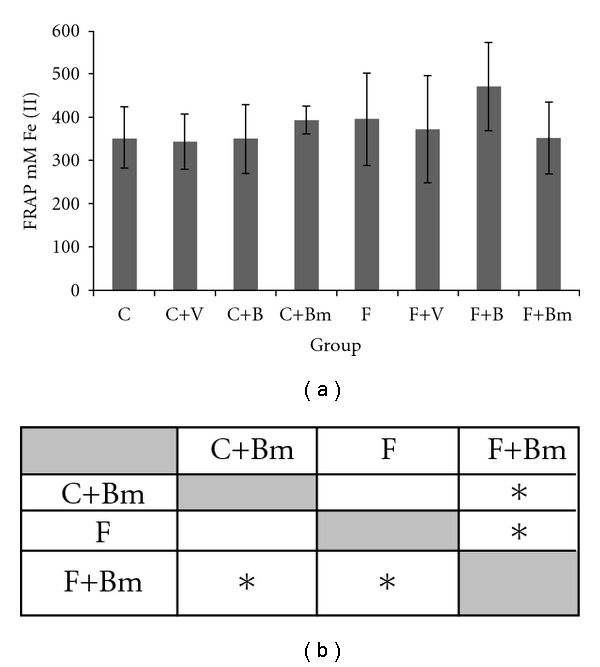
Effects of vanadium complex V, B, and Bm on plasma FRAP of Wistar rats. Data are presented as mean ± SD for *n* = 6. (b) Statistical differences between groups *P* < 0.05.

The influence of vanadium complexes on the total oxidative potential defined by the FRAP method was presented in Figure 6. It was observed that, in plasma in case of vanadium complex Bm added to the C diet, a significant growth of the FRAP value occurred in comparison to the values for the F group receiving the same vanadium complex (*P*  = 0.0209; Figure 6(a)). 

**Figure 7:**
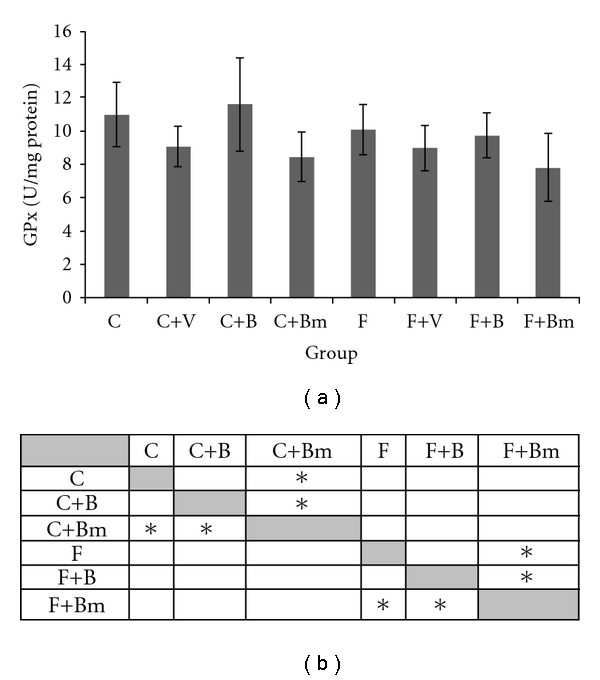
Effects of vanadium complex V, B, and Bm on plasma GPx activity of Wistar rats. Data are presented as mean ± SD for *n* = 6. (b) Statistical differences between groups *P* < 0.05.

**Figure 8:**
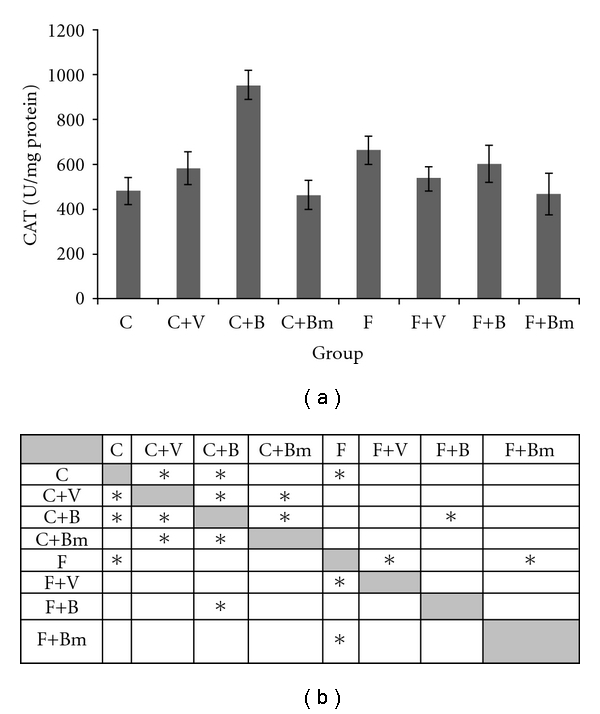
Effects of vanadium complex V, B, and Bm on plasma CAT of Wistar rats. Data are presented as mean ± SD. for *n* = 6. (b) Statistical differences between groups *P* < 0.05.

The results of GPx and CAT activity after vanadium treatment are shown in Figures 7 and 8. In the presented investigations, vanadium complexes significantly decreased glutathione peroxidase activity in plasma in all groups except the C+B group (Figure 7). In plasma GPx activity decreased from 11.01 ± 1.95 U/mg proteins value to 8.45 ± 1.47 U/mg proteins (*P*  = 0.0455; Figure 7(a)) for Bm complex in the C group and for Bm complex in the F group from 10.07 ± 1.51 U/mg proteins value to 7.81 ± 2.05 U/mg proteins (*P*  = 0.0209; Figure 7(a)).

For the next enzyme catalase, we observed higher activity in plasma for groups treated with vanadium complexes V and B in comparison to the control group C (Figure 8). In the control group C, catalase (CAT) has smaller activity 482 ± 57 U/mg proteins than those for complex V 580 ± 73 U/mg proteins and for complex B 951 ± 65 U/mg proteins. These differences were also significant (*P*  = 0.0455 and *P*  = 0.0027; Figure 8(a)). For the group F animals, plasma vanadium complexes V, B, and Bm reduced the CAT activity. Very clear response was observed in the F+Bm group where CAT activity was reduced from 662 ± 62 U/mg proteins (F group) to 467 ± 9 U/mg proteins. In the F+B group, the CAT activity was decreased to 601 ± 83 U/mg proteins and in the F+V group to 534 ± 56 U/mg proteins. Statistical significances were also calculated (*P*  = 0,0027; Figure 8(a)).

**Figure 9:**
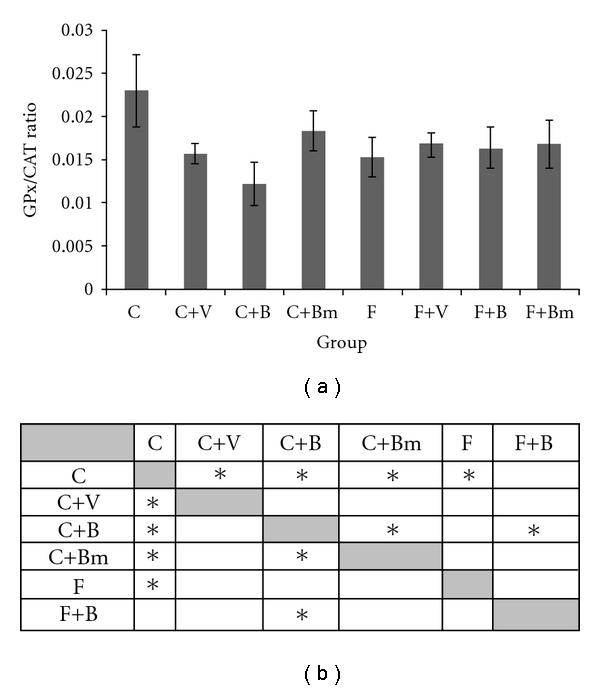
Effects of vanadium complex V, B, and Bm on plasma GPx/CAT ratio of Wistar rats. Data are presented as mean ± SD for *n* = 6. (b) Statistical differences between groups *P* < 0.05.

The changes in the relation of GPx/CAT Ratio after the application of vanadium complexes were decreased in the plasma in case of the control group C (Figure 9). Significant decrease of GPx/CAT Ratio in the C group from 22.8 ± 4.2 to 15.6 ± 1.1 in case of complex V as well as 12.2 ± 2.6 in case of complex B was recorded (*P* = 0,0027; Figure 9(a)). The decrease of GPx/CAT ratio after the application of vanadium complex Bm was noticed in the C group. Decreasing of GPx/CAT ratio in plasma from 22.8 ± 4.2 to 18.2 ± 2.3 after the application of complex Bm (*P*  = 0,0455; Figure 9(a)) was observed. Changes in GPx/CAT ratio were not observed in the F group after five weeks of adding vanadium complexes (Figure 9). The value of GPx/CAT ratio represents the ability of plasma to dissolve the peroxide of hydrogen In the control group the studied vanadium complexes accelerated the dissolution of H_2_O_2_.

## 5. Discussion and Conclusion

Many drugs and chemicals at relatively low dosages affect the metabolism of biota by altering normal enzyme activity, particularly by the inhibition of a specific enzyme. The effects could be negative and systemic [16].

The unique redox and spectroscopic properties result in metal ions and their complexes having potential medicinal applications that could be complementary to organic compounds. Recent achievements in the development of metal-based therapeutics demonstrate that this is a potentially prosperous area for inorganic chemistry and have stimulated noteworthy interest in the chemical community. 

Oxidative stress plays a foremost role in etiology of several diabetic complications [17–19]. Oxidative stress in type 2 diabetes may be the result of both antioxidant system failure and increased production of ROS. Laboratory markers for oxidative stress and measurement of total antioxidant activity of plasma are a useful tool for the qualitative and quantitative determination of free radical events. These parameters help to control the treatment of chronic diseases, including type 2 diabetes [20]. Similar results of vanadium complexes on changes in increase of body weight were obtained by Mukherjee et al. [21]. Works of other authors present lowering of cholesterol and TG in streptozotocin diabetic rats but not in normal diets [22–25]. In the literature there are no information about vanadium with fatty diets so it is difficult to discuss about obtained results. 

Vanadium complex V in the F diet had no effect on changing the concentration of UA. This suggest positive effect of vanadium before oxidative stress. In animals fed with the F diet, the addition of vanadium complexes did not cause statistically significant changes in the concentration of urea. Observed effect in plasma can suggests influence of vanadium treatment on protein metabolism and must be more examined in the future. The FRAP assay gives fast, reproducible results with plasma, with single antioxidants in pure solution and with mixtures of antioxidants in aqueous solution and was used for calibration added to plasma. On the basis of investigations presented by Russo et al. [26], the relationships of vanadium probably do not cause increased concentration of peroxides of lipids, which are natural substrata of this enzyme [26–28]. In the studied animals' plasma, the decrease of GPx activity occurred. Therefore, it can be assumed that the studied vanadium complexes do not cause changes in the concentration of the peroxides of lipids. 

The CAT plays a major role in the protection of plasma from the toxic effects of H_2_O_2_,and partially reduced oxygen species. Catalase, iron-containing enzyme (oxidoreductase) which catalyses the breakdown of H_2_O_2_ is a potentially destructive agent in cells [29]. 

In this experiment, vanadium complexes administered in small doses showed significant influence on catalase activity. Decreased CAT activity in plasma in the F+V, F+B, and F+Bm groups may be due to enzyme protein oxidation as a result of accumulation of H_2_O_2_ and other radicals. The observed decrease in CAT activity after administration of vanadium complexes may be related to oxidative inactivation of enzyme protein [30]. 

In the F group, similarly, after addition of vanadium complexes, the decrease of the GPx activity was observed. However, our results are in contrast with those of GPx activity which demonstrated that the use of vanadium complex V in the C group lowered the level of activity of GPx enzymes and raised the level of activity of CAT.

Mutual connection between catalase and glutathione peroxidase is also examined by other authors [31]. The results of those studies were divergent. Additional parameters such as dose, type of compound, duration, and others can influence results. Accordingly, further investigation of the mechanism of antioxidative defense after drug supply is required.

There are many drugs and chemicals, which are known to have adverse or beneficial effects on human enzymes and metabolic events. Inhibition of some important enzymes, which play a key role in a metabolic pathway, may lead to pathologic conditions or disorders. The toxicity of relationships of vanadium is generally low, dependent on the degree of oxidation of this element (it depends on the degree the oxidation) and the way of its application [32, 33].

Poisonings by vanadium complexes were not observed in natural endemic conditions. However, the toxicity of relationships of vanadium toxic effects were affirmed in other studies on human subjects [34]. 

In concentration, the relationships of vanadium produce symptoms of sharp poisoning, depending on the way of application, the dose, and the time of exposure of organism to the working of this element [35]. 

From the other side, vanadium and its compounds present different answer in comparable conditions. Change of oxidation state or change in ligand structure has influence on biochemical parameters and total antioxidative status. The present study show that answer can be more complicated after vanadium compounds' administration. Complexes B and Bm of vanadium are similar in structure. Addition of methyl group significantly has influence on GPx and catalase activity (Figures 8 and 9). 

The ability of vanadium complexes in inhibiting the lipid from peroxidation thereby preventing the ROS generation has restored the imbalances in the antioxidants and plasma enzymes responsible for the cell dysfunction and destruction. Vanadium has also the possibility to change the oxidative step in living organism and can have positive or negative influence on total oxidative defense. This mechanism is very variable and dependent on oxidative step, used dose, type of ligands and others. It suggests that the addition of other small function groups can change organism response on tested compound. For the better understanding of these differences, bigger experiment is necessary. This study is a first step in evaluating vanadium complexes containing fatty dietary supplements Although some significant differences were seen for the vanadium complexes analyzed in this study, any related conclusions would require additional research, including the analysis of additional vanadium complexes, the monitoring of products over time, and the evaluation of appropriate vanadium complexes' groupings.

## Figures and Tables

**Table 1:** The composition of the control diet (C) and high fat diet (F) administered to Wistar rats.

Components	Control diet (C) %	High Fatty diet (F) %
Starch	62	32
Casein	20	20
Oil	5.0	5.0
Lard	0	30
Calcium carbonate	2.8	2.8
Ca_3_(PO_4_)_2_	2.9	2.9
Lecithin	1.0	1.0
NaCl	0.3	0.3
Cellulose	4.7	4.7
Minerals and vitamins mix.	1.0	1.0
MgO	0.07	0.07
K_2_SO_4_	0.23	0.23
